# M^7^G-related LncRNAs: A comprehensive analysis of the prognosis and immunity in glioma

**DOI:** 10.3389/fgene.2022.961278

**Published:** 2022-11-16

**Authors:** Shuaishuai Wu, Augustine K. Ballah, Wenqiang Che, Xiangyu Wang

**Affiliations:** First Affiliated Hospital, Jinan University, Department of Neurosurgery, Guangzhou, China

**Keywords:** glioma, lncRNA, m 7 G, prognosis model, immune analysis, therapy

## Abstract

Today, numerous international researchers have demonstrated that N^7^-methylguanosine (m^7^G) related long non-coding RNAs (m^7^G-related lncRNAs) are closely linked to the happenings and developments of various human beings’ cancers. However, the connection between m^7^G-related lncRNAs and glioma prognosis has not been investigated. We did this study to look for new potential biomarkers and construct an m^7^G-related lncRNA prognostic signature for glioma. We identified those lncRNAs associated with DEGs from glioma tissue sequences as m^7^G-related lncRNAs. First, we used Pearson’s correlation analysis to identify 28 DEGs by glioma and normal brain tissue gene sequences and predicated 657 m^7^G-related lncRNAs. Then, eight lncRNAs associated with prognosis were obtained and used to construct the m^7^G risk score model by lasso and Cox regression analysis methods. Furthermore, we used Kaplan-Meier analysis, time-dependent ROC, principal component analysis, clinical variables, independent prognostic analysis, nomograms, calibration curves, and expression levels of lncRNAs to determine the model’s accuracy. Importantly, we validated the model with external and internal validation methods and found it has strong predictive power. Finally, we performed functional enrichment analysis (GSEA, aaGSEA enrichment analyses) and analyzed immune checkpoints, associated pathways, and drug sensitivity based on predictors. In conclusion, we successfully constructed the formula of m^7^G-related lncRNAs with powerful predictive functions. Our study provides instructional value for analyzing glioma pathogenesis and offers potential research targets for glioma treatment and scientific research.

## Introduction

Glioma is one of the most common aggressive and fatal primary tumors in the central nervous system, accounting for approximately 30% of cases (Mousavi et al., 2022). They are graded by the World Health Organization (WHO) as I to IV with increasing malignancy based on the histopathological characteristics of the tumor (Ostrom et al., 2019). Although genetic and molecular testing has brought advances in disease diagnosis, surgery, radiotherapy, and other comprehensive treatments have brought hope to patients; their prognosis is still poor. It is getting more severe economic pressure and burdening patients, their families, and society (Frances et al., 2022; Haddad et al., 2022). Thus, there is an urgent need to detect glioma-related biomarkers in our clinical care for early identification and diagnosis and to investigate new therapeutic approaches.

Although long non-coding RNA (lncRNA) is non-coding RNA that cannot be translated into protein RNA molecules, several reports have demonstrated that lncRNA regulates tumorigenesis and development (Yang et al., 2016; Chen et al., 2021). For example, *LINC01503* promotes the cancer stem cell properties of glial cells by reducing the degradation of GLI2 (Wei et al., 2022). The lncRNA *HOXA-AS2* can enhance the expression of *KDM2A*/*JAG1*, which can contribute to Treg cell proliferation and immune tolerance in gliomas and promote tumor development (Zhong et al., 2022). The downregulation of lncRNA *TTTY15*, which targets miR-4500, could regulate the proliferation and apoptosis of A172 glioma cells (Wang et al., 2022). LncRNA *IRAIN* overexpression inhibits glioma progression and temozolomide resistance by suppressing the PI3 K-related signaling pathway (Guo et al., 2022). LncRNA *KB-1460A1.5* suppresses glioma development through the miR-130a-3p feedback loop (Xu et al., 2022). Despite some progress in previous studies, few biomarkers have been studied for lncRNA prognosis to differentiate patients. Therefore, we investigated the prognostic role of m^7^G-related lncRNAs in glioma by identifying m^7^G-related DEGs in glioma in order to be able to find more useful biomarkers for glioma.

N^7^-methylguanosine (m^7^G) refers to the methylation of guanosine at the N^7^ position. m^7^G RNA modification is one of the most common posttranscriptional modifications; it is widely distributed in the 5′hat region of tRNA, rRNA, and eukaryotic mRNA and plays an essential role in gene expression, protein synthesis and transcriptional stability (Pei and Shuman, 2002; Jaffrey, 2014; Song et al., 2020). M^7^G can regulate the secondary structure of RNA or protein-RNA interaction through electrostatic and spatial effects (Furuichi, 2015). Current studies have demonstrated that almost every stage of the life cycle can be adjusted by m^7^G modifications, such as transcription (Pei and Shuman, 2002), mRNA splicing (Jiang et al., 2018), nuclear output (Lewis and Izaurralde, 1997), and translation (Marchand et al., 2018). The mutation of m^7^G methyltransferase is related to many diseases. Mutations, knockouts, and overexpression of m^7^G-related genes, such as WD repeat domain 4 (*WDR4*), lead to microcephalic primordial dwarfism (Sauna and Kimchi-Sarfaty, 2011), Nervous system damage (Lin et al., 2018), and impairment of learning and memory abilities (Pereira et al., 2009). Furthermore, *METTL1* is an author of m^7^G, essential for suppressing lung cancer cell migration through m^7^G editing on RAS and MYC driver genes (Balzeau et al., 2017; Pandolfini et al., 2019). Also, overexpression of mettl1 and bad prognosis of patients with liver cancer is associated with the downregulation of tumor suppressors in hepatocellular carcinoma (Barbieri et al., 2017; Tian et al., 2019). The tRNA N^7^-methylguanosine modification mediated by *METTL1*/*WDR4* promotes the development of squamous cell carcinoma (Chen et al., 2022). Furthermore, METTL1-m7G-EGFR/EFEMP1 axis is a precise mechanism for bladder cancer development (Ying et al., 2021). Therefore, if we want a further biological understanding of the interaction between lncRNA and cancer, we must study m^7^G modifications and explore new prognostic and therapeutic markers. In this study, we constructed a formula based on m^7^G prognosis-related lncRNAs; and verified their outstanding performance in prognosis prediction. The lncRNAs associated with glioma prognosis were also identified, which may provide potential research directions for analyzing glioma’s pathogenesis and clinical treatment**.**

## Materials and methods

### Patients and datasets

We downloaded glioma data (GBM and LGG) and normal brain tissue RNA transcriptome data from the Cancer Genome Atlas (TCGA) and the Genotype-Tissue Expression (GTEx) website (698 glioma samples and 1152 normal human brain samples, respectively). Validation data were available from the China Glioma Genome Atlas (CGGA,1018 glioma samples). Meanwhile, clinical information of glioma patients was downloaded from the TCGA and CGGA databases, and patients without follow-up data or an overall survival <30 days were excluded. Since the data in this study were obtained from public databases, ethics committee approval was not required according to the relevant regulations of the databases.

### Identify the expression of m^7^G-related genes

First, we obtained 3 genes from the published article about m^7^G (Tomikawa, 2018; Pandolfini et al., 2019; Teng et al., 2021). Then we searched for three biological pathways related to m^7^G in GSEA and extracted genes involved in each pathway. After removing duplicate genes and summarizing the above genes, we obtained 29 genes. Then, we used Wilcoxon’s method (*FDR* < 0.05, *Log*_*2*_ *FC* >1) to screen the genes with significant differences in the expression level between glioma and normal tissues based on these 29 genes. After deleting the genes with no significant differences, the remaining ones are m^7^G-related differentially expressed genes (m^7^G-related DEGs), and named them m^7^G-related genes (Supplementary Material S1). Expression of m^7^G-related DEGs samples were visualized using vioplot. Gene Ontology (GO) and Kyoto Encyclopedia of Genes and Genomes (KEGG) analysis implemented in R.

### Establishment of the risk signature

First, we performed co-expression analysis of 28 m^7^G-related genes and lncRNAs in the TCGA and GTEx glioma and normal brain tissue datasets, identifying 657 m^7^G-related lncRNAs (Pearson correlation coefficients >0.4, *p* < 0.001, Supplementary Material S2). Secondly, the prognostic relationship of m^7^G-related lncRNAs was assessed by univariate Cox regression (Supplementary Material S3). In the univariate analysis, the m^7^G-related lncRNAs with *p* < 0.01 (539 lncRNAs) were included in the least absolute shrinkage and selection operator (Lasso) regression. The results derived from Lasso regression were then incorporated into a multivariate Cox model to derive eight prognostic m^7^G-related lncRNAs and create the risk scores (RS)formula:

$$risk\;score=\overset{n}{\underset{i=1}{\sum }}coef\;m7GLncSigi\times \mathrm{EXP}\;m7GLncSigi$$

The “coef m7GLncsigi” in this “risk scores” formula represents the coefficient value, which is the regression coefficient of the 8 prognostic lncRNAs derived from the multifactorial regression analysis. The “EXP m7GLncSigi” in the formula represents the expression levels of the 8 m7G-related lncRNAs. By using the RS formula, we can get the risk value of each patient. And after getting the risk value of all patients, we can find out the median risk value of the patients. According to the median value, we can determine the level of risk of the patients.

#### Validation of the risk scoring model

Kaplan-Meier (K-M) analysis, time-dependent ROC, principal component analysis (PCA), independent prognostic analysis, nomogram (1-, 3-, and 5-year), calibration curve and the expression level of lncRNAs are used to determine the accuracy of the model. In the CGGA validation sample, we applied the same intermediate values to assess the validity and reliability of our RS formula using the same way as above. We also use the same approach to randomly divide the TCGA data into two groups for internal validation.

#### Functional annotation of prognostic m^7^G-related LncRNAs

We divided the patients into high-risk and low-risk groups based on the median risk score. GSEA (version 4.1.0, (*p* < 0.05 and FDR <0.25))was used for functional enrichment analysis (Subramanian et al., 2005). The infiltrating fraction of 16 immune cells and the activity of 13 immune-related pathways were measured by ssGSEA (Rooney et al., 2015). We also explored the relationship between risk scores and immune checkpoints in both risk groups (Yao et al., 2021).

#### Drug sensitivity correlation analysis

To find more drugs for the treatment of glioma, we focused on evaluating and predicting immune-related drugs. According to the online tool Cancer Drug Sensitivity Genomics, the IC50 of different drugs on glioma samples was predicted using the R package ‘pRRophetic’. The main use of ‘pRRophetic’ is to predict phenotypes from gene expression data (to predict clinical outcomes using Cancer Genome Project CGP cell line data), to predict drug sensitivity in external cell lines (CCLE) and also for clinical data prediction.

#### Statistical analysis

This study used R software (version 4.1.2) and GSEA software for statistical analysis. Wilcoxon test was used to identify the expression levels of m7G-related DEGs in cancer and normal tissues. Survival curves were generated using the Kaplan-Meier method and compared using the log-rank test. Cox regression and Lasso regression were used to evaluate the prognostic influences of m7G-related lncRNA features.

## Results

### Differential expression and enrichment analysis of m^7^G-related genes

**FIGURE 1:**
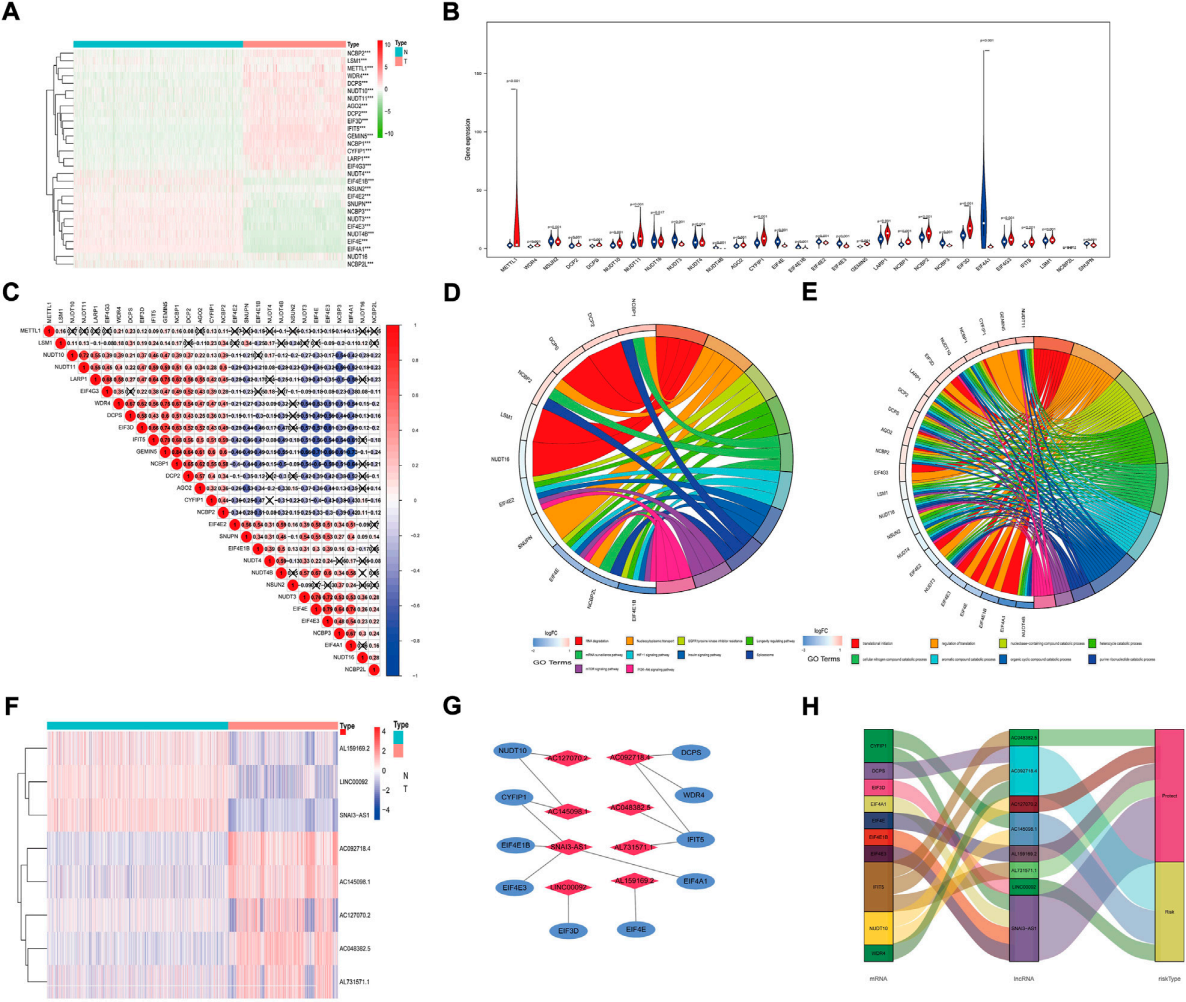
Expression of m^7^G-related mRNAs and prognostic m^7^G-Related lncRNAs. **(A)** Heat Map shows the expression levels of eight m^7^G-related mRNAs. **(B)** The vioplot shows the differentially m^7^G-related mRNAs. Blue represents normal sample, and red represents the glioma sample. **(C)** Spearman correlation analysis of m^7^G-related mRNAs. **(D)** KEGG circle diagram of m^7^G-related DEGs. **(E)** GO circle diagram of m^7^G-related DEGs. **(F)** The expression levels of eight prognostic m^7^G-Related lncRNAs. **(G)** The co-expression network of prognostic m^7^G-related lncRNAs. **(H)** Sankey diagram of prognostic m^7^G-Related lncRNAs. lncRNAs, long non-coding RNAs; N, normal; T, tumor.

After analysis, we found that 28 m7G-related genes were significantly differentially expressed between glioma and normal tissues (Figure 1A). Specifically, *NUDT11*, *IFIT5*, *GEMIN5*, *METTL1*, *CYFIP1*, *NCBP1*, *WDR4*, *NUDT10*, *EIF3D*, *LARP1*, *DCP2*, *DCPS*, *AGO2*, *NCBP2*, *EIF4G3* and *LSM1* were highly expressed in tumor samples (*p* < 0.001). *NSUN2*, *NUDT4*, *EIF4E2*, *SNUPN*, *NCBP3*, *NUDT3*, *EIF4E3*, *EIF4E*, *NCBP2L*, *EIF4E1B*, *EIF4A1* and *NUDT4B* were lowly expressed in tumor samples (*p* < 0.001). The expression levels of *NUDT16* were not significantly different (*p* = 0.517) (Figure 1B). In addition, to further understand the intrinsic association between the 28 m^7^G-related genes, we also performed a correlation analysis. The results showed that the positive correlation between *GEMIN5* and *NCBP1* was the most significant, and the negative correlation between *GEMIN5* and *EIF4A1* was the most significant (Figure 1C). The above results suggest some interaction between m^7^G-related genes in glioma. Then, KEGG pathway analysis showed that m^7^G-related DEGs were mainly enriched in RNA degradation, Nucleocytoplasmic transport, *EGFR* tyrosine kinase inhibitor resistance, Longevity regulating pathway, mRNA surveillance pathway, *HIF-1* signalling pathway, Insulin signalling pathway, Spliceosome, mTOR signalling pathway, *HIF-1* signalling pathway and PI3K-Akt signalling pathway (Figure 1D). GO analysis showed that DEGs were mainly enriched in the regulation of translation, nucleobase-containing compound catabolic process, heterocycle catabolic process, *Etc.* (Figure 1E).

### Screening prognostic m^7^G-related LncRNAs

We identified 658 lncRNAs associated with m^7^G-related genes. Univariate Cox regression analysis showed that 540 lncRNAs were linked to patient prognosis. One hundred thirty-two were considered risk lncRNAs with HR > 1, while 408 were protective lncRNAs with HR < 1. After Lasso regression, 28 m7G-associated lncRNAs were identified. Finally, multivariate Cox regression identified 8 lncRNAs with the best prognostic correlation (*AC048382.5*, *AC127070.2*, *AL159169.2*, *AL731571.1*, *SNAI3-AS1*, *AC092718.4*, *AC145098.1*, *LINC00092*) (Supplementary Material S4). The expression levels of the eight prognostic m^7^G-related lncRNAs are shown (Figure 1F). We used the Cytoscape and ‘galluvial’ R packages to visualize the lncRNAs. The co-expression network contained 14 lncRNA-mRNA pairs (Figure 1G, R2>0.4, *p* < 0.001). *SNAI3-AS1* was co-expressed with four related genes (*EIF4A1*, *EIF4E3*, *EIF4E1B*, and *CYFIP1*), AC092718.4 was co-expressed with three related genes (*IFIT5*, *DCPS*, and *WDR4)*, and *AC145098.1* was co-expressed with two related genes (*CYFIP1* and *NUDT10*), *AC127070.2* co-expressed with *NUDT10*, *AC048382.5* and *AL731571.1* both co-expressed with *IFIT5*, *LINC00092* co-expressed with *EIF3D* and *AL159169.2* co-expressed with *EIF4E*. *AC048382.5*, *AC127070.2*, *AL159169.2*, *AL731571.1*, and *SNAI3-AS1* were protective factors, while *AC092718.4*, *AC145098.1*, and *LINC00092* were risk factors (Figure 1H).

### Development and validation of prognostic models

**FIGURE 2:**
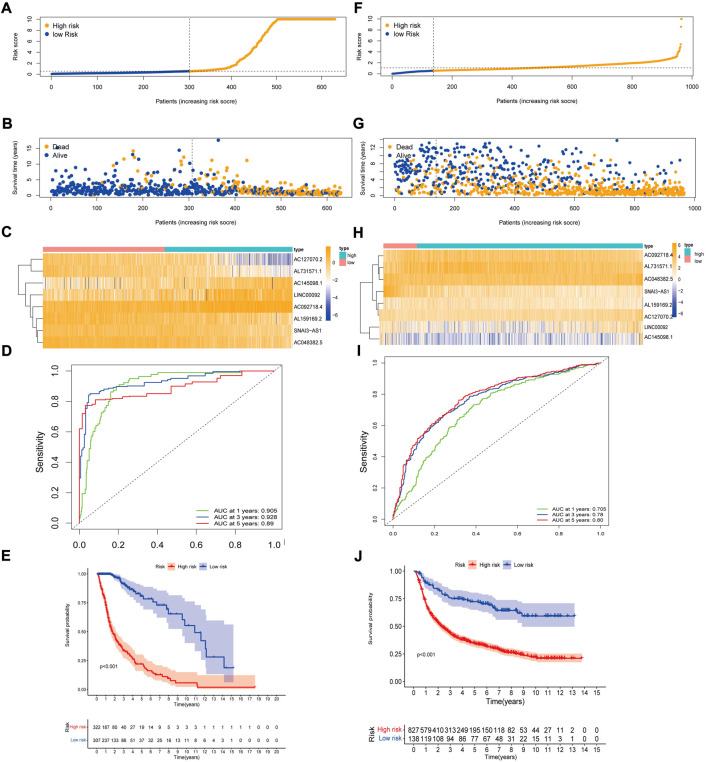
Construction and validation of the 8 prognostic m^7^G-related lncRNAs signature for survival prediction. **(A)** Distribution of RS in TCGA. **(B)** Survival time and status of patients in TCGA. **(C)** Heatmap of prognostic m^7^G-related lncRNAs of RS in TCGA. **(D)** ROC curve for TCGA. **(E)** KM curve for TCGA. **(F)**Distribution of RS in CGGA. **(G)**Survival time and status of patients in CGGA. **(H)**Heatmap of m^7^G-related lncRNAs of RS in CGGA. **(I)**ROC curve for CGGA. **(J)** KM curve for CGGA.

Based on the above eight lncRNAs, we constructed a prognostic model and calculated the risk score for each patient using the risk score model. The risk score formula worked as follows: risk score =(0.620302782 × AC092718.4 expression) + (0.492232265 × LINC00092 expression) + (0.724211508 × AC145098.1 expression) + (-0.922536934 × SNAI3-AS1 expression) + (-0.922536934 × AC048382.5 expression) + (-0.846208391 × AC127070.2 expression) + (-0.924348861 × AL731571.1 expression) + (-0.807182397 × AL159169.2 expression). After obtaining a risk score for each patient, the patients were divided into two groups based on the median risk score: a high-risk group and a low-risk group (Figure 2A). We found that more and more patients died as the risk score increased (Figure 2B). Figure 2C showed eight prognostic m7G-related lncRNAs involved in two groups by heat map. The ROC curve area showed the excellent predictive capability of the model based on eight survival-related lncRNAs. In the TCGA data, the AUC values were 0.905, 0.928, and 0.89 at 1, 3, and 5 years, respectively (Figure 2D). According to KM analysis, patients with high RS had worse survival rates than those with low RS (Figure 2E).

Using the same cut-off from the TCGA data for the CGGA validation data, it was possible to distinguish the high-risk group from the low-risk group. However, the number of patients in the low-risk group was significantly lower (Figure 2F). CGGA patients showed that high-risk patients are positively associated with poor prognosis (Figure 2G). The expression of prognostic m^7^G-related lncRNAs in CGGA resembled that in TCGA samples (Figure 2H). In the CGGA sample, the AUC values were0.705, 0.78, and 0.80 at 1, 3, and 5 years, respectively (Figure 2I). KM analysis performed on CGGA data showed the same results as TCGA data (*p* < 0.001, Figure 2J). The validation results in the two validation datasets of TCGA also demonstrate the excellent predictive power of the model (Supplementary Material S5).

### Validation of PCA analysis and the expression of prognostic LncRNAs

**FIGURE 3:**
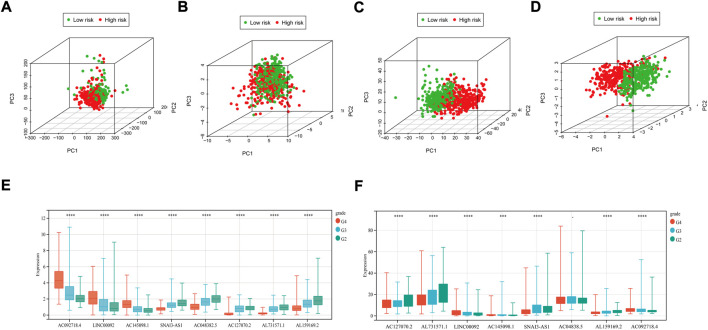
PCA maps of the TCGA glioma dataset show the distribution of patients based on the **(A)** whole genome; **(B)** m^7^G-related gene sets; **(C)**m^7^G-related lncRNAs; and **(D)** prognostic m^7^G-related lncRNAs. Red: high-risk; Green dots: low-risk. Expression profile of 8 prognostic m^7^G-related lncRNAs with different glioma grades. **(E)**m^7^G-related lncRNAs expression with different glioma grades in TCGA datasets. **(F)** prognostic m^7^G-related lncRNAs expression with different glioma grades in CGGA datasets. (G2: WHO II, G3: WHO III, G4: WHO IV). *p < 0.05, **p < 0.01 and ***p < 0.001.

The distribution of patients based on whole genes, m^7^G-related genes, m^7^G-related lncRNAs, and prognostic m^7^G-related lncRNAs was visualized using PCA plots. The results showed that m^7^G survival-associated lncRNA showed the best results (Figures 3A–D). High- and low-risk patients can be distributed in different quadrants according to the RS of prognostic m^7^G-related lncRNAs.

We evaluated the expression levels of m^7^G-related lncRNAs in the TCGA dataset. We found that all genes differed significantly in different grades (Figure 3E), and all but one of the genes had similar trends across stages (Figure 3F). In TCGA and CGGA datasets, the same trend of gene expression was shown with increasing tumor grade.

### Validation of the correlations between clinical variables and risk score

**FIGURE 4:**
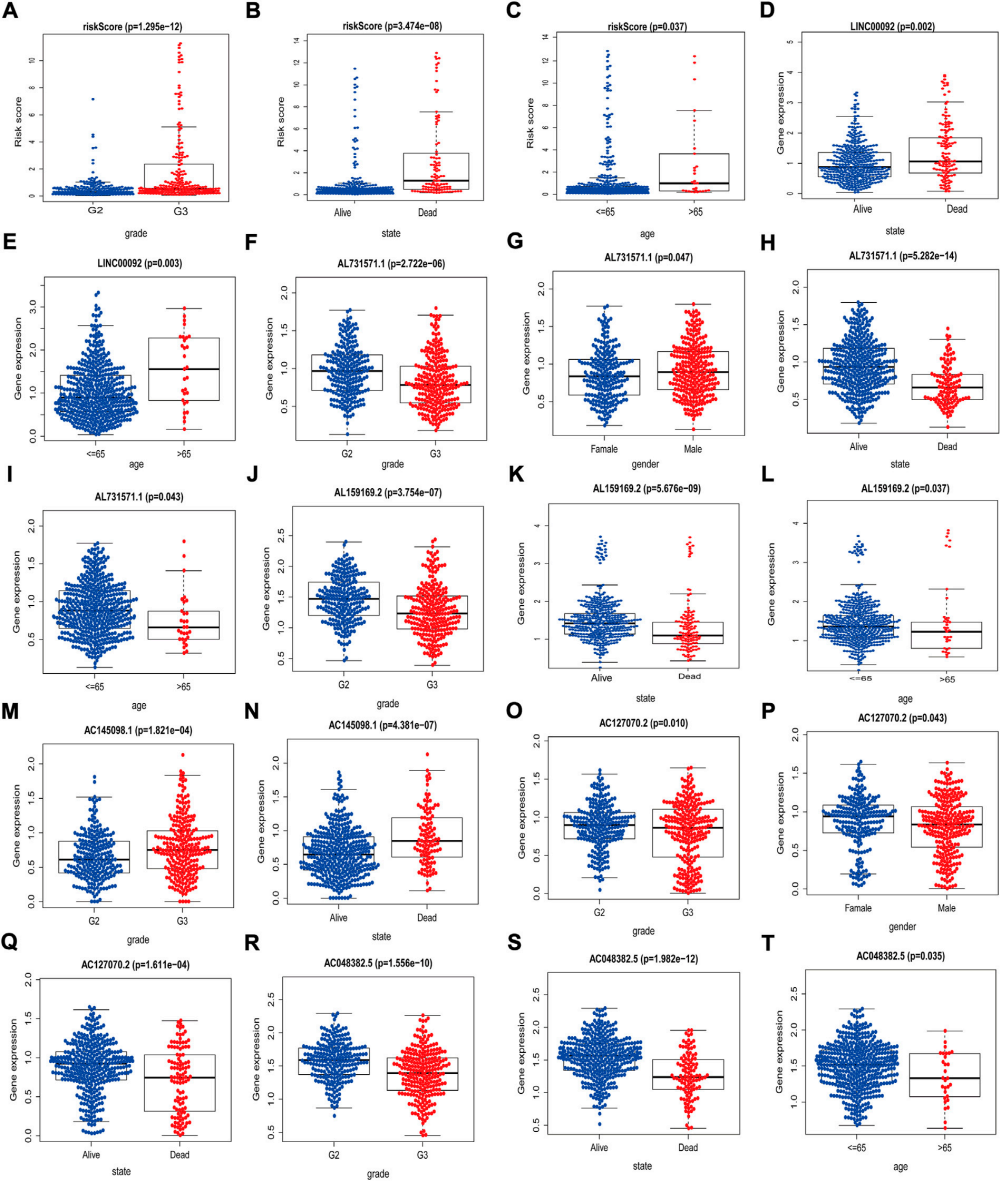
Associations between risk scores/related lncRNAs and clinical features. **(A–C)** Association between risk score and gender, state, and age. **(D–E)** Association between *LINC00092* expression level and state, age. **(F–I)** Association between *AL731571.1* expression level and grade, gender, state and age. **(J–L)** Association between *AL159169.2* expression level and grade, state and age. **(M–N)** Association between *AC145098.1* expression level and grade and state. **(O–Q)** Association between *AL127070.2* expression level and grade, gender and state. **(R–T)** Association between *AC048382.5* expression level and grade, state and age.

Using TCGA data, we analyzed the correlations between these clinical variables and the eight lncRNAs risk scores. The risk scores were correlated with age, survival status, and tumor stage; *AC048382.5* was associated with age, survival status, and stage; *AC127070.2* was correlated with survival status, sex, and stage; *AC145098.1* was correlated with survival status and stage; *AL159169.2* was correlated with age, survival status, and stage; *AL731571.1* was correlated with age, survival status, gender, and staging; and *LINC00092* was associated with age and survival status. (Figure 4). The above results showed that our screened m^7^G-related lncRNAs had the excellent predictive ability.

### Development and validation of nomogram

**FIGURE 5:**
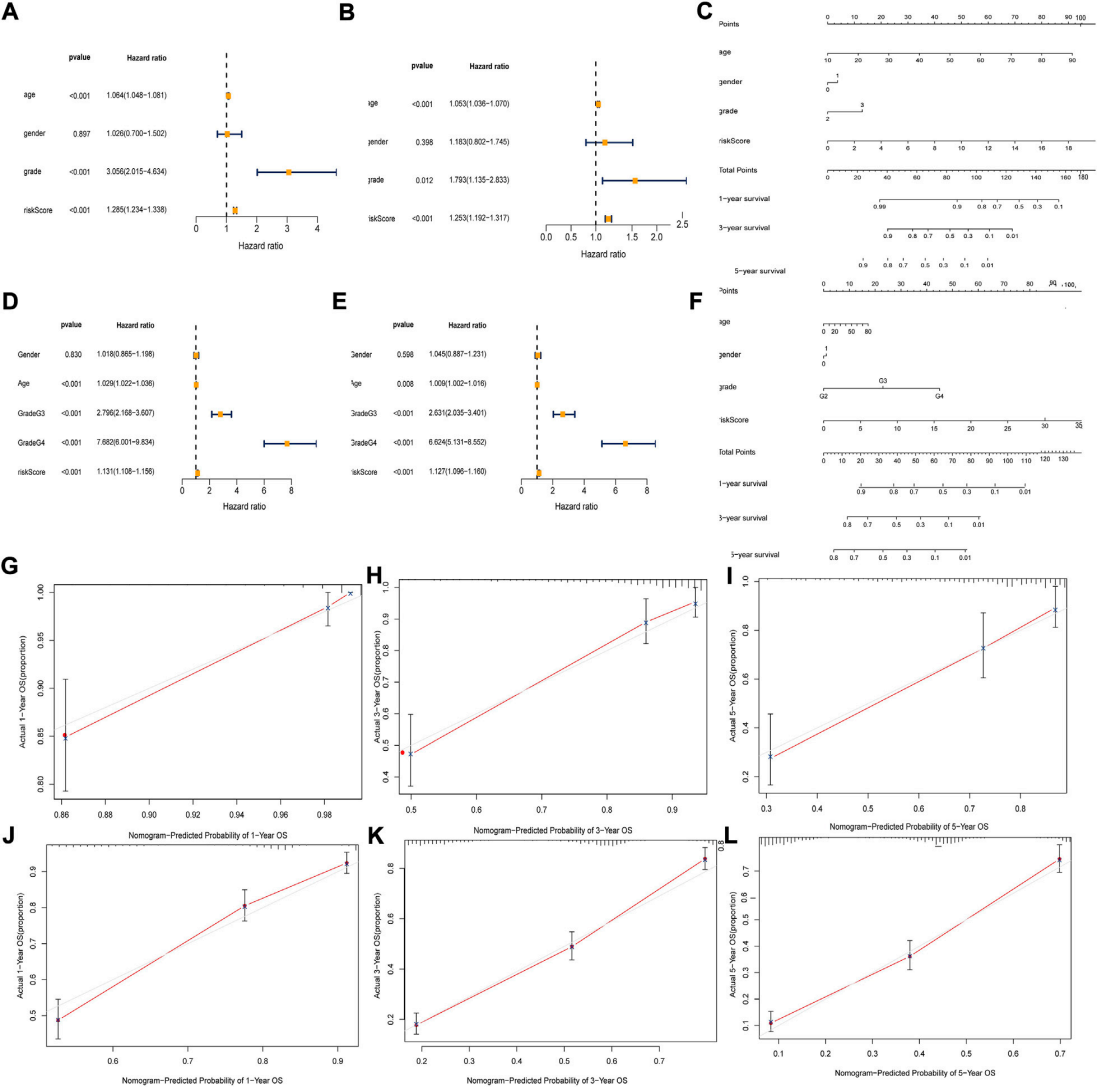
Independent prognosis analysis of risk score. **(A and D)** Univariate COX Forest plot of the risk score in TCGA and CGGA. **(B and E)** Multivariate COX Forest plot of the risk score in TCGA and CGGA. **(C and F)** Nomogram based on prognostic features in TCGA and CGGA. **(G–I)** Calibration plots of the nomogram for predicting the probability of OS at 1, 3, and 5 years in the TCGA. **(J–L)** Calibration plots of the nomogram for predicting the probability of OS at 1, 3, and 5 years in the CGGA.

In TCGA and CGGA data, we analyzed the independent prognostic factors of glioma patients by Cox regression. Univariate and multivariate Cox regression analyses showed that risk score was an independent predictor (HR = 1.253, 95% CI:1.192–1.317, *p* < 0.001; HR = 1.127, 95% CI:1.096–1.160, *p* < 0.001) of OS in glioma patients (Figure 5A, B,D,E). We constructed a column line plot containing clinicopathological variables and risk scores to facilitate clinical work (Figure 5C,F). The calibration curves showed good agreement between actual OS and predicted survival rates (Figures 5J–L).

### Functional annotation of m^7^G-related lncRNAs

**FIGURE 6:**
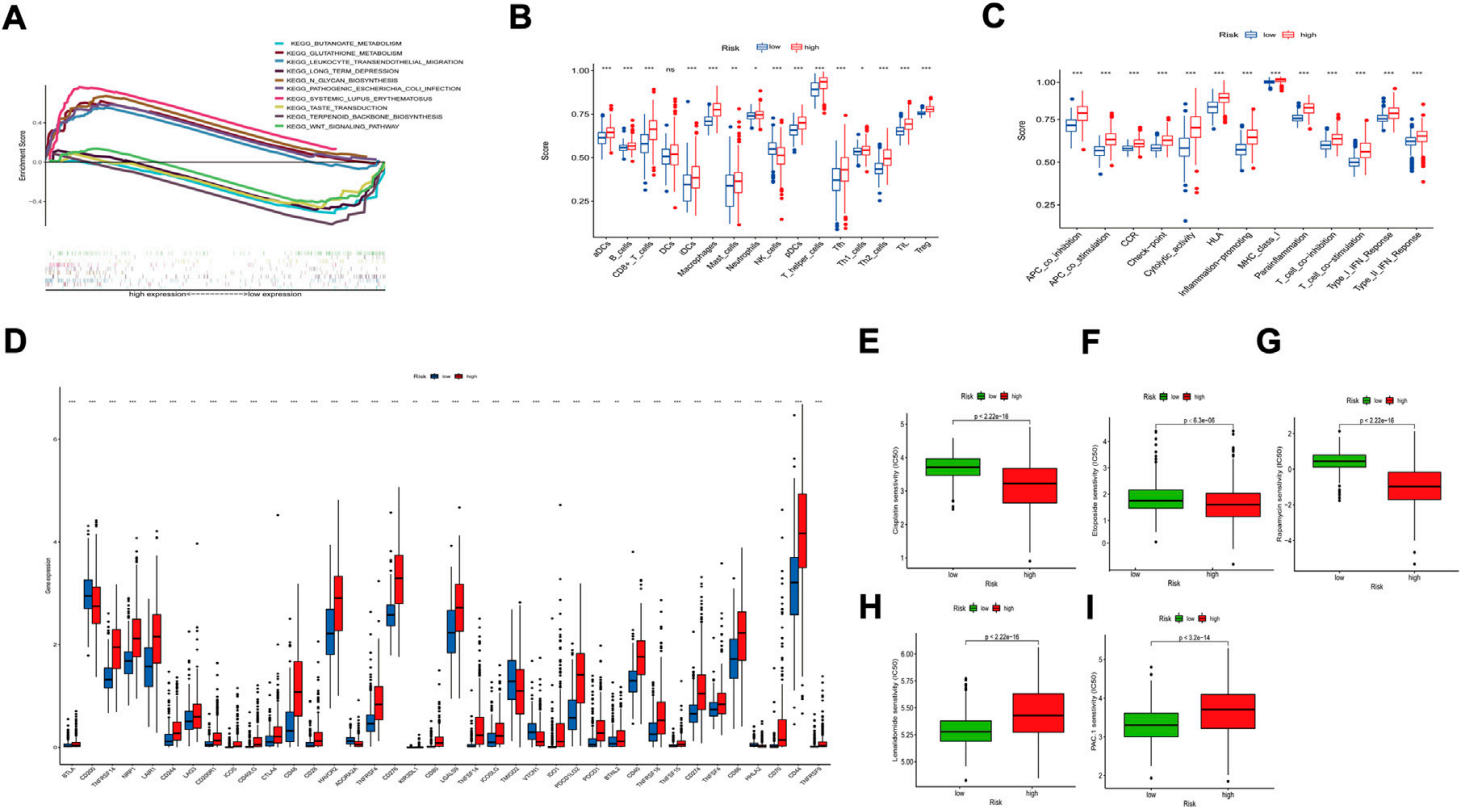
Functional enrichment analysis of 8 prognostic m^7^G-related lncRNAs. **(A)** KEGG analysis of 8 prognostic m^7^G-related lncRNAs. **(B)** The infiltration levels of 16 immune cells. **(C)** The correlation between the predictive signature and 13 immune-related functions. **(D)** Expression of immune checkpoints. aDCs, activated dendritic cells; iDCs, immature dendritic cells; NK, natural killer; pDCs, plasmacytoid dendritic cells; Tfh, T follicular helper; Th1, T helper type 1; Th2, T helper type 2; TIL, tumor-infiltrating lymphocyte; Treg, T regulatory cell; APC, antigen-presenting cell; CCR, chemokine receptor; HLA, human leukocyte antigen; MHC, major histocompatibility complex; IFN, interferon. *p < 0.05; **p < 0.01; ***p < 0.001; ns, non-significant. Comparison of treatment drugs sensitivity between high- and low-risk groups. **(E–I)** IC50 of Cisplatin, Etoposide, Rapamycin, Lenalidomide, PAC.1 in high and low-risk groups. IC50, half-maximal inhibitory concentration.

We used GSEA to investigate further the differences between the two subgroups for eight m^7^G-related lncRNAs. In KEGG analysis, the main added functions were systemic lupus-erythematosus, n-glycan-synthesis, and glutathione-metabolism. Decreased functions were wnt-signalling-pathway, taste-transduction, and terpenoid-backbone-biosynthesis (Figure 6A). Most of these pathways are mainly responsible for immune-related diseases and metabolic pathways. So, this suggests that poor prognosis in high-risk patients is likely to be closely related to tumor immune-related pathways.

### Investigation of immune-related pathways

We quantified the enrichment scores of ssGSEA by measuring the immune cell subpopulations and related pathways to investigate further the correlation between risk scores and immune cells and functions. In the high-risk group, we found a significant rise in most cells (B cells, CD8^+^ T cells, DCs, Tregs, *etc.*) (Figure 6B). T-cell-co-inhibition, APC-co-stimulation, CCR, T-cell-co-stimulation, and type I IFN response were higher in the high-risk group than in the low-risk group (Figure 6C). The above results suggest that the high-risk group’s immune function was more active. We also compared the analysis of differences in immune checkpoint expression between the two groups because of the importance of checkpoint-based immunotherapy (Figure 6D).

### Correlation of predictive features between drug sensitivities

We also analyzed the correlation between predictive characteristics and tumor immune-related drugs. The results found lower IC50 of Cisplatin, Etoposide, and Rapamycin in the high-risk group and higher IC50 of Lenalidomide and PAC-1 in the high-risk group. (Figures 6E–I), which helps to explore individualized treatment regimens suitable for high-risk patients.

## Discussion

Glioma is a common brain tumor, accounting for 78% of primary malignant brain tumors in the brain, and its overall prognosis has been poor. Therefore, exploring the early diagnosis of glioma and accurately predicting the prognostic markers is of crucial clinical significance (Linzey et al., 2019). Many studies have shown the critical role of m^7^G in cancer development, mainly focusing on the regulation of tumor cell genesis and progression, but few investigations on cancer prognosis (Orellana et al., 2021; Rong et al., 2021; Xia et al., 2021). Several studies have recently emerged by constructing mRNA and lncRNA predictive signatures associated with glioma autophagy, pyrogenesis, m^6^A, and ferrogenesis can be used to predict the prognosis of glioma patients (Maimaiti et al., 2022) (Zhou et al., 2021a) (Guan et al., 2021) (Shi et al., 2022). However, the study of prognostic m7G-related lncRNAs in glioma has not been reported. Therefore, we purpose to investigate the prognostic role of m^7^G-related lncRNA in glioma and provide a new approach for the future clinical treatment of glioma.

This study first obtained 28 DEGs associated with N^7^-methylguanosine. Then, KEGG analysis showed that DEGs were mainly enriched in RNA degradation, nucleocytoplasmic transport, mRNA surveillance pathway, HIF-1, mTOR, and HIF1-PI3K-Akt signaling pathway. GO analysis showed that DEGs were primarily enriched in the translational initiation activity, regulation of translation, RNA 7−methylguanosine, *etc.* Existing studies have modified mRNA by adding an m^7^G 5′ cap to protect mRNA from premature degradation (Kasprzyk and Jemielity, 2021). EGFR plays a crucial role in the METTL1-m7G axis in bladder cancer (Ying et al., 2021). Upregulated WDR4 expression increases m^7^G methylation levels in hepatocellular carcinoma (Xia et al., 2021). Hickey et al. reported that m^7^G-MP, the cap analog, is a potent and specific inhibitor of eukaryotic translation (Hickey et al., 1976). The above results suggest that m^7^G-related genes maybe participate in cancer development through various pathways such as transcription and translation. However, further studies are needed to explore the function of m^7^G-related genes in glioma.

In addition, there are pieces of evidence that lncRNAs play an essential part in cancer (Ho et al., 2022; Liang et al., 2022; Zhang et al., 2022). *SNAI3-AS1*, an m^7^G prognosis-associated lncRNA, is an important tumor modifier in hepatocellular carcinoma tumor progression (Li et al., 2020). Recently, it has been reported that autophagy-related lncRNA features can accurately predict the prognosis of glioma patients (Maimaiti et al., 2022). Ferroptosis-associated lncRNAs can also predict the prognosis of glioma patients (Shi et al., 2022). Therefore, it is important to identify the predictive value of m^7^G-related lncRNAs in glioma patients and could provide potential directions for future experimental studies of m^7^G and clinical studies of glioma. In this study, we identified 8 prognostic m^7^G-related lncRNAs (*AC048382.5*, *AC127070.2*, *AL159169.2*, *AL731571.1*, *SNAI3-AS1*, *AC092718.4*, *AC145098.1*, *LINC00092*) for establishing prognostic model. We also found mRNAs (*EIF4A1*, *EIF4E3*, *EIF4E1B*, *CYFIP1*, *DCPS*, *WDR4*, *NUDT10*, *IFIT5*, *EIF3D*, *EIF4E*) were significantly co-expressed with these lncRNAs. Among them, eIF4E binds the 7-methyl-GTP portion of the 5′ cap structure of cytoplasmic mRNA and plays a part in translation initiation and regulation (Merrick and Pavitt, 2018). Additional studies have found that DCPS acts on m^7^G through mRNA decay (Ng et al., 2015). *WDR4* undergoes a malignant transformation of cells through overexpression of m^7^G (Orellana et al., 2021). *EIF4* acts as a cap-binding protein to enhance m^7^G cap stabilization of transcripts and plays an important role in malignancy through upregulation (Culjkovic-Kraljacic et al., 2020). In conclusion, the above reports provide evidence for our related studies on N^7^-methylguanosine. In analyzing two databases with the same median, we found that the number of deaths increased as the risk score increased. The 5-year AUC values (AUC = 0.89, AUC = 0.80) in both TCGA and CGGA data demonstrated the success of the model construction in predicting the prognosis of glioma patients. Furthermore, eight lncRNAs expression in different grades of glioma, the correlation between risk scores and clinical characteristics also increases their predictive power.

Then, GSEA shows that the high-risk group mainly enriched systemic lupus-erythematosus, n-glycan-biosynthesis, glutathione-synthesis, and leukocyte-transendothelial migration. N^7^-methyladenosine, a common methylation modification of RNA, plays an essential role in autoimmune diseases like RA and SLE (Agris et al., 1992; Zhou et al., 2021b). N-glycan plays a significant part in breast and oral cancers (Hirano and Furukawa, 2022; Wu et al., 2022). Glutathione affects tumor progression by altering oxidative stress sensitivity in astrocytic tumors (Moreira Franco et al., 2021). Increased expression of lymphocyte-specific protein 1 (LSP1) will cause leukocyte migration and inhibition of the immune microenvironment in GBM (Cao et al., 2020). The above results suggest that the occurrence and development of gliomas are also most likely to be closely related to immune-related pathways. The ssGSEA results showed a significant rise in most cells (macrophages, CD8^+^ T cells, mast cells, Tregs, *etc.*) in the high-risk group. Some of the above findings have been confirmed by studies. For example, CD8^+^T-cell infiltration is associated with poor prognosis in patients with BC (Hou et al., 2020; Liu et al., 2020). High infiltration of tumour-associated macrophages was associated with low-grade glioma and thyroid cancer (Ryder et al., 2008; Li et al., 2022). The number of mast cells was positively linked to poor prognosis in patients with prostate cancer (Zhang et al., 2020).

The degree of MC infiltration in mice and human gliomas is proportional to the malignancy of the tumor (Polajeva et al., 2011; Polajeva et al., 2014). The ratio of high neutrophils to lymphocytes predicts a poorer OS in BC patients (Tan, 2017). Pathological grading of gliomas is positively correlated with infiltrating neutrophils (Khan et al., 2020). Increased infiltration of Tregs indicates a poor prognosis in patients with hepatocellular carcinoma (Tu et al., 2016). lncRNA *HOXA-AS2* promotes Treg proliferation and immune tolerance through the miR-302A/*KDM2A* axis to promote glioma progression and poor prognosis (Zhong et al., 2022). Increased Treg and MDSC in mouse gliomas can lead to a decrease in overall survival (Zhai et al., 2021). We found higher HLA and type I IFN response scores in the high-risk group, except for increased tumor immune cell infiltration. Thus, decreased antitumor immunity in high-risk groups may be responsible for poor prognosis. We found significant differences in immune checkpoint expression between the high-risk and low-risk groups. We also studied the sensitivity of immune-related drugs among patients and found that high-risk patients may be sensitive to Cisplatin, Etoposide, and Rapamycin and resistant to Lenalidomide, PAC-1. This implies that high-risk groups may benefit from treatment with multiple immune-related drugs. We hope the above study provides a basis for precise, individualized treatment of glioma patients.

However, our study has some limitations. In the first place, we only used CGGA and CGGA database data for verification and still required external data to test the applicability of predicted signatures. Next, the mechanism of action of m^7^G-related lncRNAs in glioma needs to be further validated experimentally.

## Conclusion

We successfully built a formula for m^7^G-related lncRNAs with powerful predictive functions and screened lncRNAs with prognostic values. These studies add some instructional value to glioma etiopathogenesis and clinical treatment analysis. And these m^7^G-related lncRNAs may become new biomarkers and are expected to provide new ideas for glioma therapeutic approaches.

## Data Availability

The datasets presented in this study can be found in online repositories. The names of the repository/repositories and accession number(s) can be found in the article/Supplementary Material.
