# Supplementary figures and images for: M7G-related LncRNAs: A comprehensive analysis of the prognosis and immunity in glioma
Source: Front Genet. 2022 Nov 16;13:961278. doi: 10.3389/fgene.2022.961278 (PMC9708876; doi:10.3389/fgene.2022.961278)

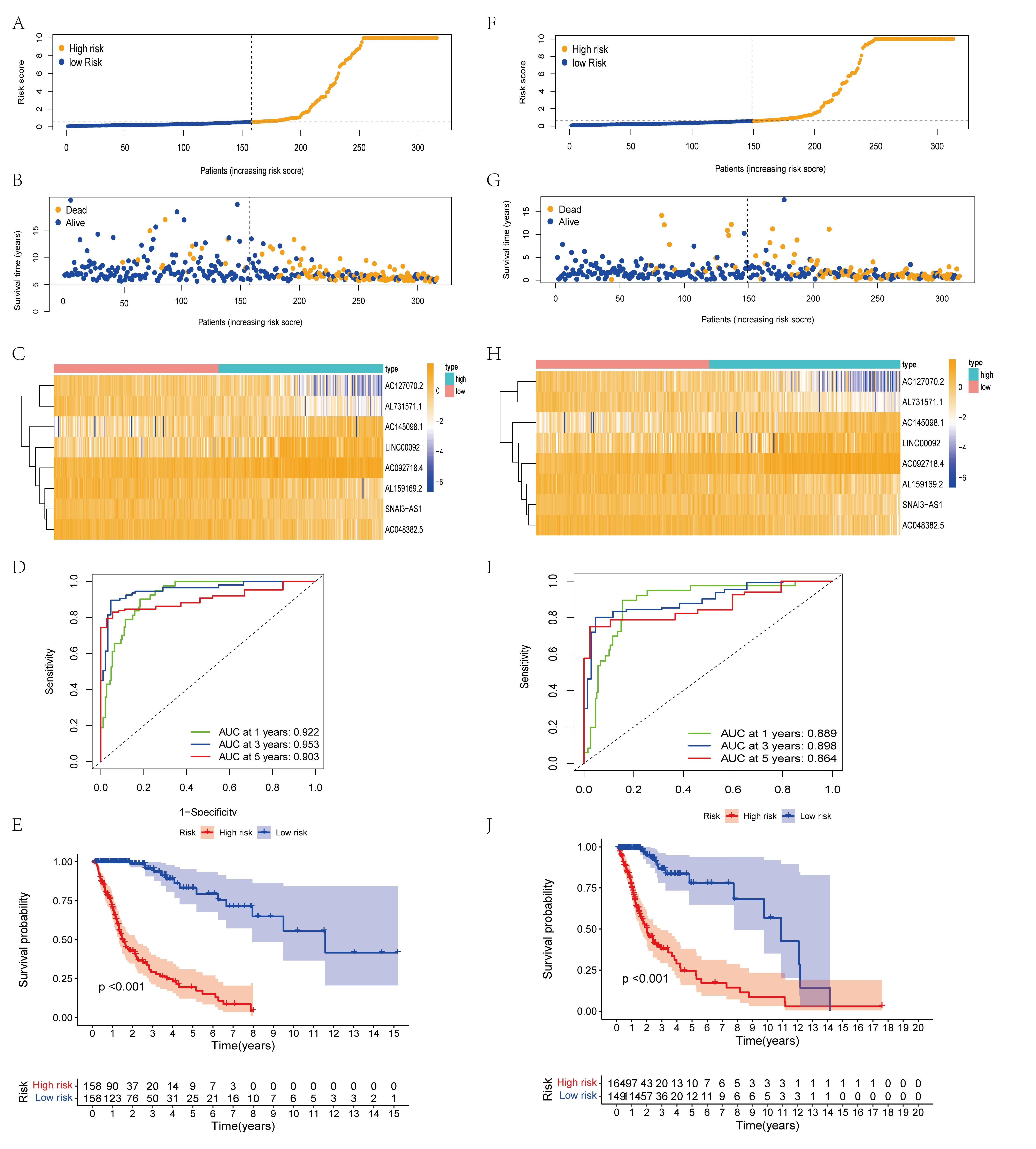

Supplement: Supplementary file 3 [file Image1.JPEG]
